# Prevalence of tobacco smoking and associated factors among adults in Ethiopia: a systematic review and meta-analysis

**DOI:** 10.3389/fpubh.2024.1353033

**Published:** 2024-07-03

**Authors:** Chala Daba, Amanuel Atamo, Sisay Abebe Debela, Mengesha Dagne, Belay Desye, Mesfin Gebrehiwot

**Affiliations:** ^1^Department of Environmental Health, College of Medicine and Health Sciences, Wollo University, Dessie, Ethiopia; ^2^Department of Public Health, College of Health Science, Salale University, Fitche, Ethiopia

**Keywords:** tobacco smoking, addiction, adult population, meta-analysis, Ethiopia

## Abstract

**Introduction:**

The public health concern of tobacco smoking is more prevalent in low- and middle-income countries including Ethiopia. Various studies have investigated tobacco smoking in various parts of Ethiopia. However, the findings have been inconsistent and characterized by significant variability. Besides, there is no nationally representative data on the subject, which could deter the design of effective intervention strategies to reduce tobacco-related problems. Therefore, this study aimed to estimate the pooled prevalence of tobacco smoking and associated factors among adults in Ethiopia.

**Methods:**

The study was conducted based on the Preferred Reporting Items for Systematic Reviews and Meta-Analysis Protocols Guideline. A detailed search was conducted from international databases including PubMed, Cochrane Library, Science Direct, CINAHL, African Journals Online, HINARI, Global Health, and Google Scholar. The extracted data was analyzed using STATA 14 software. A random-effects model was used to estimate the effect size. The Egger regression test and I^2^ statistics were used to determine potential publication bias and heterogeneity among the reviewed articles, respectively.

**Results:**

A total of 32 studies with 69,897 study participants were included in this systematic review and meta-analysis. The pooled prevalence of lifetime tobacco smoking among adults in Ethiopia was 16.0% (95% confidence Interval (CI): 13.6–18.39) and there was significant heterogeneity among the included studies (I^2^ = 99.1%, *p* < 0.001). Male adults were three times more likely to smoke tobacco as compared with females [OR = 3.22 (95% CI: 2.06–5.03)]. Being an alcohol user [OR = 3.78 (95%CI: 1.27–11.24)] and having tobacco-smoking friends [OR = 7.21 (95%CI: 5.56–9.35)] are potential determinant factors for tobacco smoking.

**Conclusion:**

The pooled prevalence of lifetime and current tobacco smoking among adults in Ethiopia was high, which calls for urgent intervention. Therefore, prioritization of tobacco control strategies, such as creating awareness about the public health importance of tobacco smoking, can help prevent and mitigate the effects of tobacco smoking. Alcohol control law enforcement should also be strengthened.

## Introduction

Tobacco smoking is one of the causes of mortality and morbidity worldwide (1). It is the second significant cause of death globally. Every 6.5 s, one tobacco user dies as a result of a tobacco-related disease (2). According to the World Health Organization (WHO), annually, 8 million deaths are reported worldwide as a result of tobacco smoking (3); 6 million are direct smokers (4, 5). It has been confirmed that tobacco smoking can cause various types of cancers (6), chronic obstructive pulmonary disease, and lung disease. For instance, 90% of all lung cancer deaths in the United States were due to tobacco smoking (7). This is associated with the fact that tobacco contains up to 7,000 health-threatening chemicals (8). Beyond mortality and morbidity, the use of tobacco adds a burden to the national economy by increasing global health expenditures by about 6% and other indirect costs (9). According to the WHO report, the cost of smoking alone is estimated to be US$ 1.4 trillion, or 1.8% of the global GDP (10).

Over 80% of the global 1.3 billion smokers are found in low- and middle-income countries, where the burden of tobacco-related illness and death is significant (1). For instance, the prevalence of tobacco smoking was 60.2% in Bangladesh (11), 30.1% in Cameron (12), 20.2% in India (13), 14.7% in Malaysia (14), 13.7% in Nigeria (15), 12.4% in Yemen (16), and 10.7% in Pakistan (17). In Ethiopia, around 2.9 million adults are cigarette smokers. Of these, one-third of adults are exposed to secondhand smoke in public places (14), which is a significant cause of premature morbidity and mortality.

Numerous studies have been conducted to assess the prevalence of tobacco smoking and associated factors in Ethiopia (18–49). However, the study reports are inconsistent and characterized by significant variability [e.g., 2.4% (33) to 45.3% (28)], which could negatively influence the design of effective intervention strategies. Moreover, although a systematic review and meta-analysis is conducted among specific groups of the population (e.g., students) (14), there is no nationwide study assessing the pooled prevalence of tobacco smoking among the general adult population in Ethiopia. Therefore, this systematic review and meta-analysis aimed to estimate the pooled prevalence of tobacco smoking and identify factors contributing to tobacco smoking among adults in Ethiopia. The findings from this meta-analysis would generate evidence that will be important inputs for tobacco smoking prevention and control program planners, policymakers, and health service providers to design and implement evidence-based interventions to reduce the burden of tobacco-related mortality and morbidity in the country and other similar settings.

## Methods and materials

### Protocol registration

The protocol for this systematic review has been registered in the International Prospective Registry of Systematic Review (PROSPERO) with a specific registration number CRD42023415610.

### Searching strategies

This meta-analysis followed the Preferred Reporting Items for Systematic Reviews and Meta-Analysis (PRISMA) guideline (50). Studies were searched through PubMed/Medline, Cochrane Library, Science Direct, CINAHL, African Journals Online, HINARI, Global Health, and Google Scholar. In addition, digital libraries were searched to identify grey literature.

Endnote software was used to collect, organize, and remove the duplications of search outcomes. The search was made using the search terms: “prevalence,” “proportion,” “magnitude,” “incidence,” “tobacco smoking,” “cigarette smoking,” “smoking,” “tobacco,” “cigarette” “substance use” “factors,” “determinants,” “predictors,” “factors associated,” “associated factors,” “risk factors,” “adult,” and “Ethiopia.” All key terms were searched by a combination of Boolean operators “AND” or “OR” as appropriate, and the search was carried out from April 1 to May 15, 2023, by three authors independently (CD, AA, and BD).

### Inclusion and exclusion criteria

Population: this systematic review and meta-analysis includes studies conducted among tobacco-smoking adults in Ethiopia. Exposure: an individual adult who experienced tobacco smoking whereas an individual adult who did not tobacco smoking was considered as a comparison. Outcome: studies assessed tobacco smoking as a primary outcome. All studies conducted with observational studies design (cohort, cross-sectional, and case–control) and institutional and community-based studies were also included in systematic review and meta-analysis. Besides, studies published from 2000 to 15 May 2023 in the English language were also included in the meta-analysis. However, qualitative studies, unretrievable studies, editorial letters, studies with poor methodological quality, and studies that did not report the outcome of interest were excluded from the meta-analysis.

### Outcome measurement

The primary outcome of the study was the pooled prevalence of tobacco smoking among adults in Ethiopia, calculated by dividing the number of smokers by the total sample size and multiplying by 100. Additionally, the study aimed to identify the factors linked to tobacco smoking in the form of a log odds ratio.

### Data extraction

After searching for relevant studies from the aforementioned electronic databases, they were exported to Endnote X20 (51) and any duplicates were removed. Three authors (CD, BD, and MG) independently extracted all the required data from the included studies by using a standardized data extraction template. The data extraction template consisted of various study details, such as the author’s name, region, publication year, study area/region, study population, study setting, study design, sample size, response rate, and tobacco smoking prevalence (current and lifetime prevalence). In the case of any disagreements during data extraction, other authors (AA, MD, and SAD) resolved them.

### Quality assessment

After duplicate files were removed, four reviewers (CD, MG, BD, and SAD) screened the relevant studies for inclusion. The Joana Brigg Institute (JBI) critical appraisal checklist for prevalence studies was employed to evaluate the quality of each article (52) and those studies scored more than 50% were included for analysis (53, 54). Each study’s quality was assessed independently out of 100% by four authors (CD, BD, MG, and SAD). If any discrepancies arose during the quality assessment, the mean score was calculated from the results of all reviewers to resolve the differences.

### Statistical analysis

The extracted data was exported into STATA/ SE version-14 statistical software for analysis (55). The level of heterogeneity among the included studies was statistically evaluated using the Higgs I^2^ test, with values greater than 75% considered high heterogeneity (56). DerSimonian and Liard’s method of random-effects model at a *p*-value less than 0.05 with a 95% CI was used to estimate the pooled prevalence of tobacco smoking using a forest plot. A *p*-value of less than 0.05 was considered indicative of the presence of heterogeneity (57).

Subgroup analysis was also conducted by characteristics of the study, such as region (Oromia, Amhara, Tigray, SNNPR (Southern Nation, Nationality, and People Region), all regions, or others), study setting (institutional or community), sample size (large—≥ 600 or small—<600), study population (students or community), and year of publication (before 2015 or 2015 and after). The different factors associated with tobacco smoking were presented using odds ratios (ORs) with 95% CI. Moreover, univariate meta-regression was carried out to identify the source of variations among studies that exhibited high heterogeneity.

Additionally, publication bias was assessed using a funnel plot and Egger’s test method with a significance level of *p* < 0.05 (58). Likewise, Duval and Tweedie’s ‘trim and fill’ analysis was also conducted to adjust the effect of publication bias among the studies included. A sensitivity analysis was also performed to assess the influence of a single study on the pooled prevalence estimates.

## Results

### Study selection

**Figure 1:**
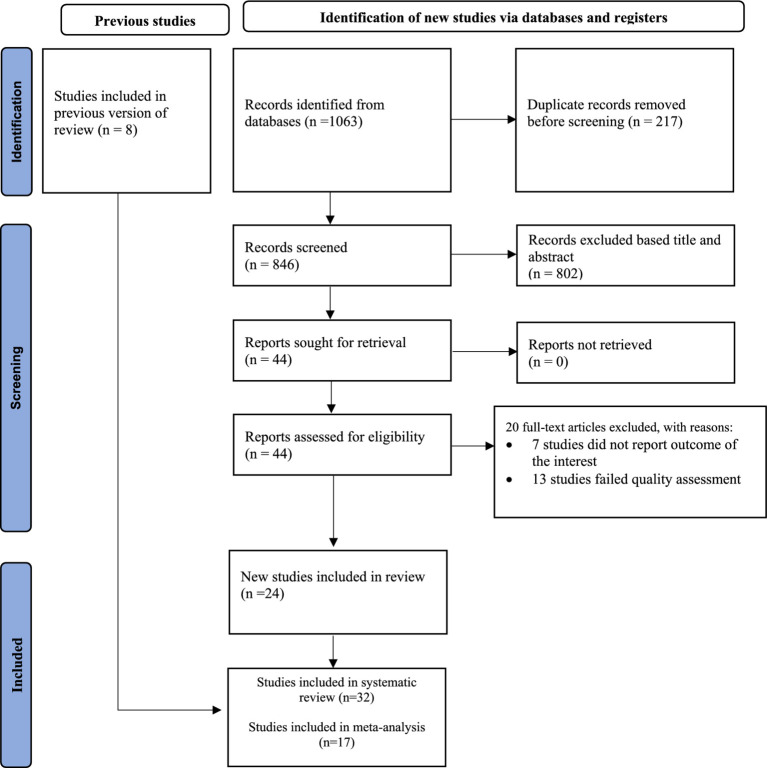
PRISMA flow diagram of the included studies for the systematic review and meta-analysis of the prevalence of tobacco smoking and associated factors among adults in Ethiopia, 2023.

After conducting the electronic database search, a total of 1,063 studies were identified. Out of these, 802 articles were excluded based on their titles and abstracts. Besides, 20 articles were also excluded due to poor quality and did not report the outcome of the interest. Finally, 32 full-text articles were found eligible for this systematic review (Figure 1).

### Characteristics of the included studies

A total of 32 articles were reviewed to estimate the pooled prevalence of tobacco smoking among adults in Ethiopia. The highest prevalence of tobacco smoking was reported in a study conducted in the Oromia region (45.3%) (28) while the lowest (2.4%) was reported in a study conducted among all regions of Ethiopia (33). The total number of study participants included in the systematic review and meta-analysis was 69,897. In this meta-analysis, eight studies were conducted in the SNNPR (20, 26, 31, 34, 38–40, 46), seven in the Oromia region (19, 22, 24, 25, 28, 42, 49), three in Amhara region (18, 35, 48), three in Tigray region (27, 29, 32), three in all regions of Ethiopia (33, 37, 41), three in Addis Ababa (23, 45, 47), two in Somalia region (21, 44), and one each in Harari region (43), Benishangul Gumiz (30) and Dire-Dawa city administration (36). Although attempts were made to search cohort, case–control, and cross-sectional studies, we found only cross-sectional studies (Table 1).

**Table 1:** A descriptive summary of 32 studies included estimating the pooled prevalence of tobacco smoking and associated factors among adults in Ethiopia, 2023.

Authors	Year of publication	Region	Study population	Methods of data collection	Study design	Sample size	Response rate (%)	Lifetime prevalence (%)	Current prevalence (%)	Quality score (%)
Gutema et al. (31)	2021	SNNPR	Community	Community	Cross-sectional	3,346	99.3	20.2	17.1	87.5
Mekiso et al. (39)	2022	SNNPR	Community	Community	Cross-sectional	591	93.8	31.0	NA	81.2
Roble et al. (44)	2021	Somali	Community	Community	Cross-sectional	341	96.9	NA	21.1	87.5
Desalegn et al. (24)	2021	Oromia	Patient	Institution	Cross-sectional	515	98.3	22.3	NA	81.2
Hagos et al. (32)	2016	Tigray	Student	Institution	Cross-sectional	271	100	11.4	5.0	75.0
Telayneh et al. (48)	2021	Amhara	Student	Institution	Cross-sectional	605	96.7	NA	6.8	75.0
Seid et al. (47)	2021	Addis Ababa	Student	Institution	Cross-sectional	383	97.7	9.6	6.4	62.5
Mengesha et al. (41)	2022	All region	Community	Community	Cross-sectional	10,150	100	5.0	3.7	75.0
Lakew et al. (37)	2015	All region	Community	Community	Cross-sectional	30,625	100	4.1	NA	75.0
Reda et al. (42)	2013	Oromia	Community	Community	Cross-sectional	548	91.0	NA	28.0	87.5
Reda et al. (43)	2012	Harari	Student	Institution	Cross-sectional	1,721	91.1	12.2	4.2	75.0
Kebede (35)	2002	Amhara	Teacher	Institution	Cross-sectional	181	75.0	28.2	13.3	62.5
Kassa et al. (34)	2014	SNNPR	Student	Institution	Cross-sectional	586	99.3	14.8	7.5	62.5
Dereje et al. (22)	2014	SNNPR and Oromia	Student	Institution	Cross-sectional	1,673	98.2	28.6	17.2	62.5
Eticha et al. (27)	2014	Tigray	Student	Institution	Cross-sectional	193	100	NA	29.5	75.0
Lodebo et al. (38)	2017	SNNPR	Community	Community	Cross-sectional	640	100	NA	23.6	75.0
Deressa et al. (23)	2011	Addis Ababa	Student	Institution	Cross-sectional	622	78.0	9.0	1.8	87.5
Alebachew et al. (19)	2019	Oromia	Student	Institution	Cross-sectional	251	98.8	39.5	35.1	75.0
Rudatsikira et al. (45)	2007	Addis Ababa	Student	Institution	Cross-sectional	1,868	NA	15.1	2.9	62.5
Hirpha et al. (33)	2023	All region	Student	Institution	Cross-sectional	3,355	97.0	4.7	2.4	81.2
Etu et al. (28)	2017	Oromia	Community	Community	Cross-sectional	634	100	NA	45.3	87.5
Gedif et al. (30)	2019	Benishangul Gumiz	Community	Community	Cross-sectional	1,588	100	15.8	NA	75.0
Gebreslassie et al. (29)	2013	Tigray	Student	Institution	Cross-sectional	756	98.7	9.5	9.3	87.5
Schoenmaker et al. (46)	2005	SNNPR	Community	Community	Cross-sectional	3,019	62.0	5.8	4.4	62.5
Tesfaye et al. (49)	2014	Oromia	Student	Institution	Cross-sectional	1,022	98.3	22.0	10.8	75.0
Adere et al. (18)	2017	Amhara	Student	Institution	Cross-sectional	655	89.7	7.9	6.4	75.0
Bago (20)	2017	SNNPR	Student	Institution	Cross-sectional	336	92.3	20.6	NA	81.2
Banti et al. (21)	2017	Somali	Student	Institution	Cross-sectional	600	92.3	NA	14.5	75.0
Kumburi et al. (36)	2017	Dire-Dawa	Student	Institution	Cross sectional	930	75.1	43.5	41.2	62.5
Mekonen et al. (40)	2017	SNNPR	Student	Institution	Cross-sectional	725	97.0	5.7	NA	75.0
Dida et al. (25)	2014	Oromia	Student	Institution	Cross-sectional	603	97.9	13.1	4.6	75.0
Duko et al. (26)	2019	SNNPR	Student	Institution	Cross-sectional	564	94.0	11.0	9.4	81.2

NA, Not available.

### Prevalence of tobacco smoking

**Figure 2:**
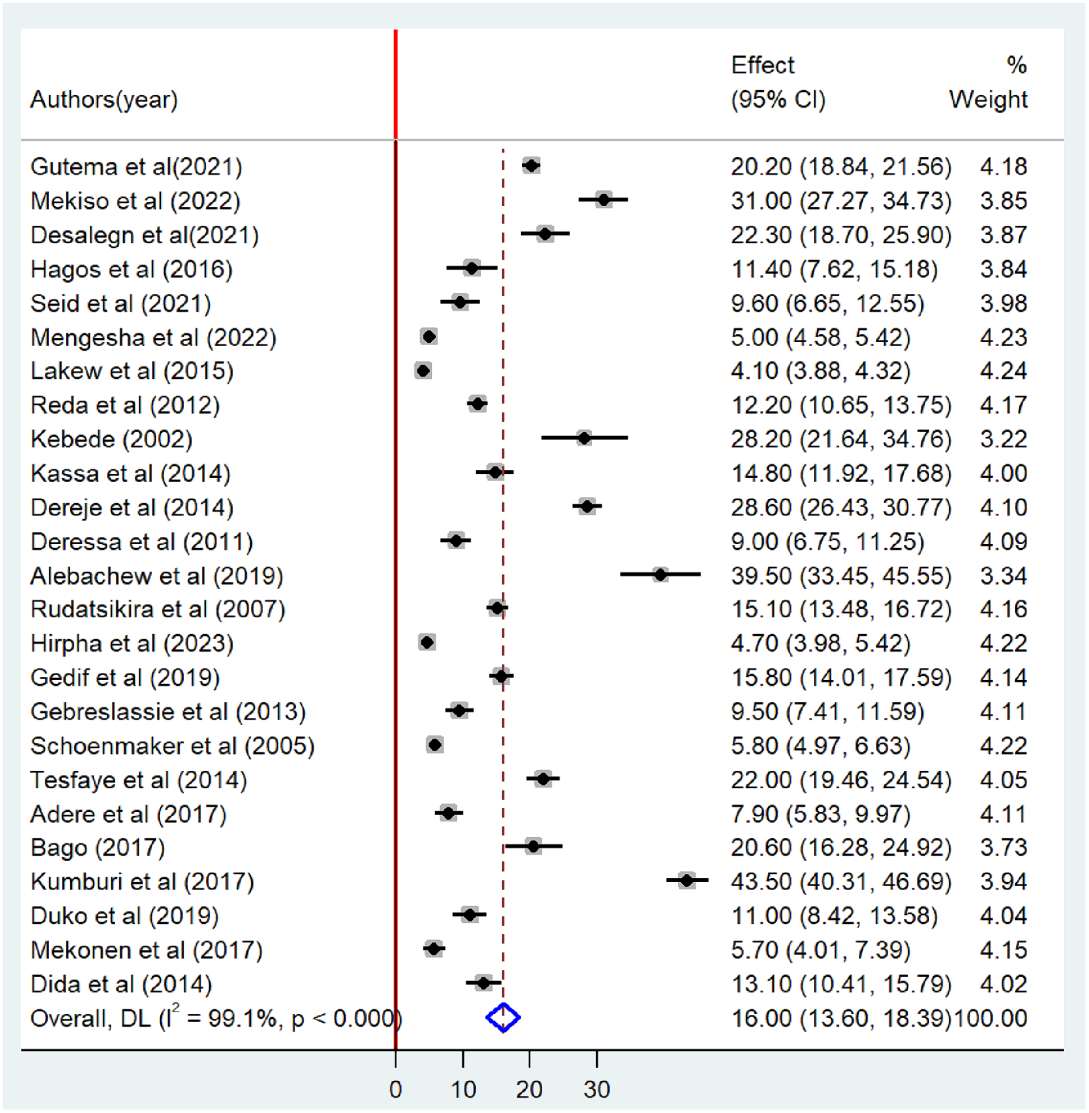
Forest plot showing the pooled prevalence of lifetime tobacco smoking among adults in Ethiopia, 2023.

**Figure 3:**
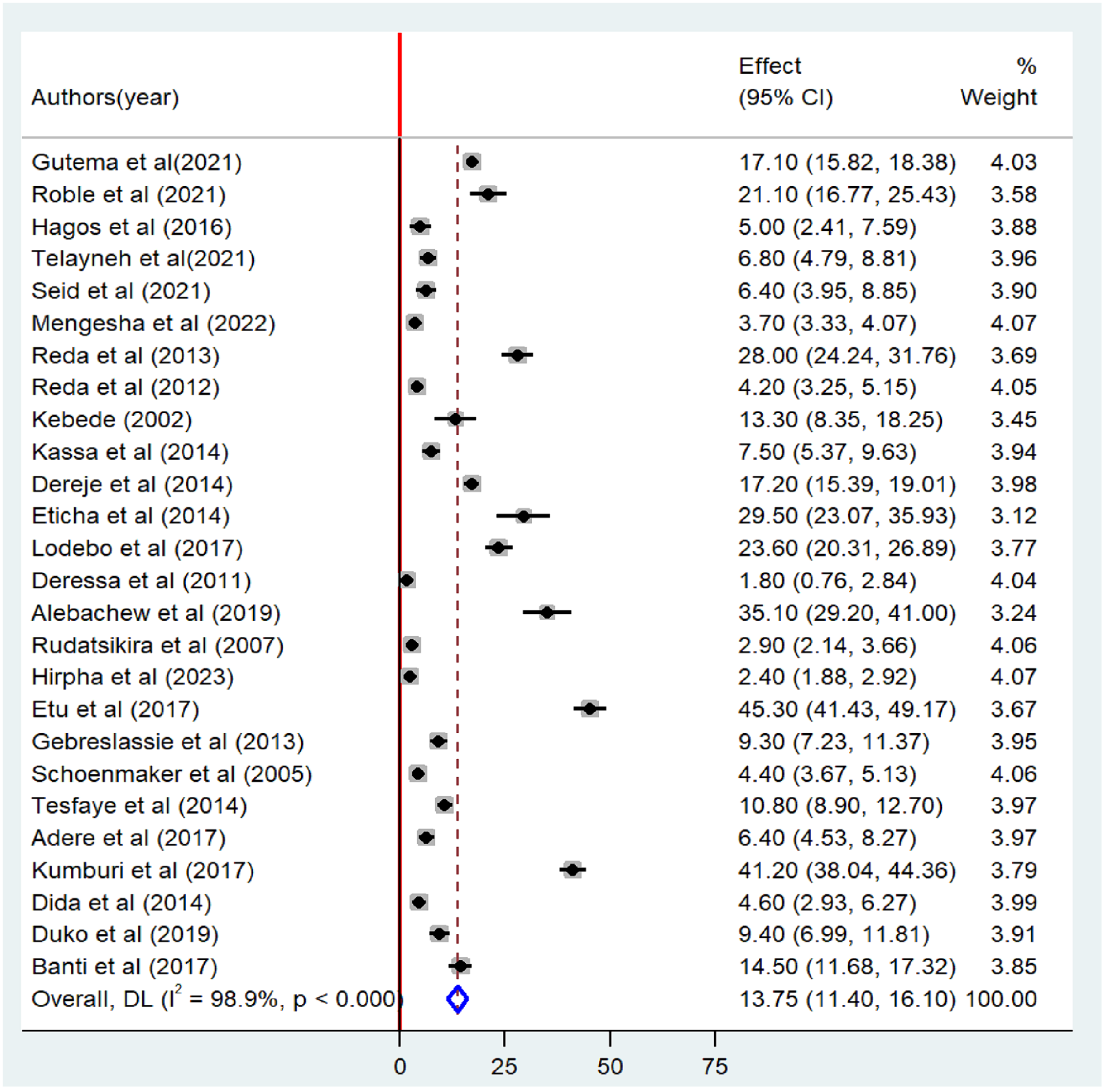
Forest plot of the pooled prevalence of current tobacco smoking among adults in Ethiopia, 2023.

In this meta-analysis, 25 and 26 studies were included to estimate the pooled prevalence of lifetime and current tobacco smoking, respectively. The pooled prevalence of lifetime tobacco smoking among adults was 16.0% (95% CI: 13.6–18.39). A random-effects model revealed that the included articles have high heterogeneity (I^2^ = 99.1%; *p* < 0.001) (Figure 2). Similarly, the pooled prevalence of current tobacco smoking was 13.7% (95% CI: 11.40–16.10), with significant heterogeneity (I^2^ = 98.9%; *p* < 0.001) (Figure 3).

### Publication bias

**Figure 4:**
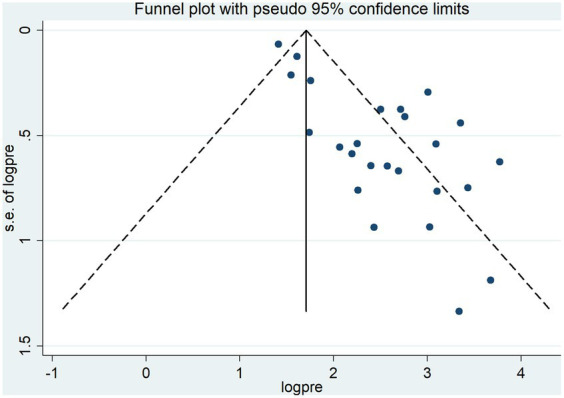
Funnel plot of the pooled lifetime prevalence of tobacco smoking among adults in Ethiopia, 2023.

**Figure 5:**
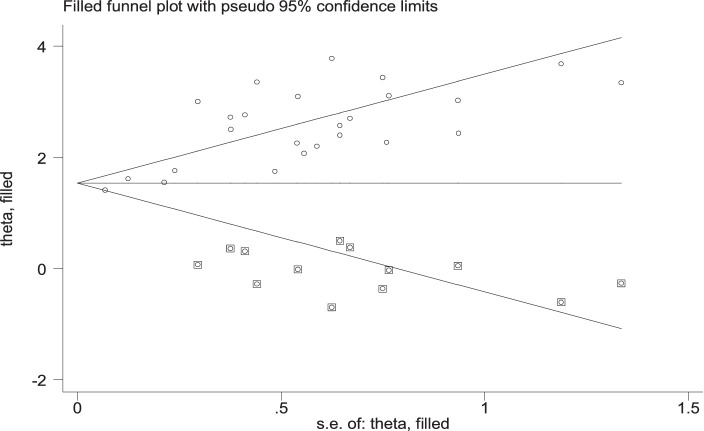
“Trim and fill” analysis funnel plot.

Publication bias was subjectively evaluated by using a funnel plot. The finding indicated that there is asymmetrical distribution of the studies, which suggests the presence of publication bias (Figure 4). The Egger test statistics also revealed the presence of statistically significant publication bias (*p* < 0.001). Duval and Tweedie’s “trim and fill” analysis indicates significant variation in the newly estimated pooled odds ratio (the adjusted point estimate) [OR = 1.68, (95% CI: 1.36–1.99)] as compared to the initial or observed point estimate [OR = 2.42, (95% CI: 2.11–2.73)] (Figure 5).

### Sensitivity analysis

**Figure 6:**
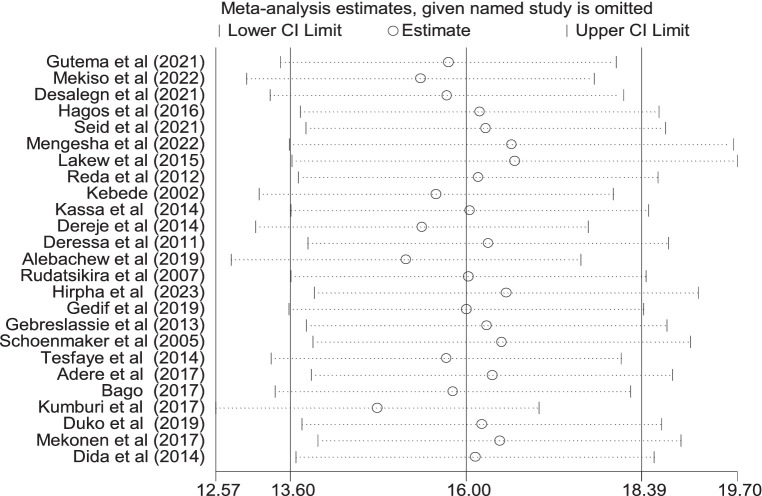
Sensitivity analysis of the pooled prevalence of lifetime cigarette smoking among adults in Ethiopia, 2023.

The findings of sensitivity analysis suggest no evidence of a single study’s effect on the overall pooled prevalence (Figure 6).

### Subgroup analysis

To identify the source of heterogeneity among the included research articles, sub-group analysis was carried out based on the region where the studies were conducted, study population, year of publication, study setting, and sample size. Even though heterogeneity was not resolved among the included articles, the pooled prevalence of lifetime tobacco smoking was relatively higher among students than the general population [16.2 (95% CI: 11.94–20.45)]. In addition, a high prevalence of tobacco smoking was observed in the studies done in the Oromia region as compared to other regions [24.7 (95%CI: 17.87–31.71)], with significant heterogeneity (I^2^ = 96.3; *p* < 0.001). Similarly, the prevalence of tobacco smoking was high among institutional-based studies [15.9 (95%CI: 13.59–18.39)] as compared with community-based studies [13.1 (95%CI: 9.64–16.60)], with statistically significant heterogeneity (I^2^ = 99.4; *p* < 0.001) (Table 2). In addition, a univariate meta-regression was conducted using the study population, sample size, and study year as factors. However, neither of them was found to be a statistically significant source of heterogeneity (Table 3).

**Table 2:** Subgroup analysis of the pooled prevalence of tobacco smoking among adults in Ethiopia, 2023.

Subgroup	Number of studies	Pooled prevalence (95% CI)	Heterogeneity	
			*I*^2^ (%)	*p*-value
By study population				
Community	8	14.5 (12.29–18.90)	99.3	< 0.001
Students	17	16.2 (11.94–20.45)	98.7	< 0.001
By study year				
Before 2015	10	15.6 (10.94–20.29)	98.5	< 0.001
2015 and after	15	16.2 (13.36–19.11)	99.2	< 0.001
By region				
Tigray	2	9.9 (8.11–11.77)	0.00	0.389
Amhara	2	17.8 (−2.09–37.69)	97.0	< 0.001
Oromia	5	24.7 (17.87–31.71)	96.3	< 0.001
SNNPR	7	15.5 (9.15–21.77)	98.8	< 0.001
All region	3	4.6 (3.91–5.23)	86.4	< 0.001
Other region *	6	17.5 (10.39–24.54)	98.6	< 0.001
By study setting				
Institution	19	15.9 (13.59–18.39)	98.6	< 0.001
Community	6	13.1 (9.64–16.60)	99.4	< 0.001
By sample size				
Large (≥600)	16	13.7 (11.00–16.43)	99.3	< 0.001
Small (<600)	9	20.7 (14.81–26.51)	95.7	< 0.001

*: Somali, Harari, Dire Dawa, Addis Ababa, Benishangul Gumuz.

**Table 3:** Univariate meta-regression analysis to identify factors associated with the heterogeneity of the prevalence of tobacco smoking in Ethiopia, 2023.

Variables	Coefficient	*p*-value
Study setting	0.2945229	0.437
Sample size	−0.9491036	0.174
Study year	−0.3927522	0.307

### Factors associated with tobacco smoking

**Figure 7:**
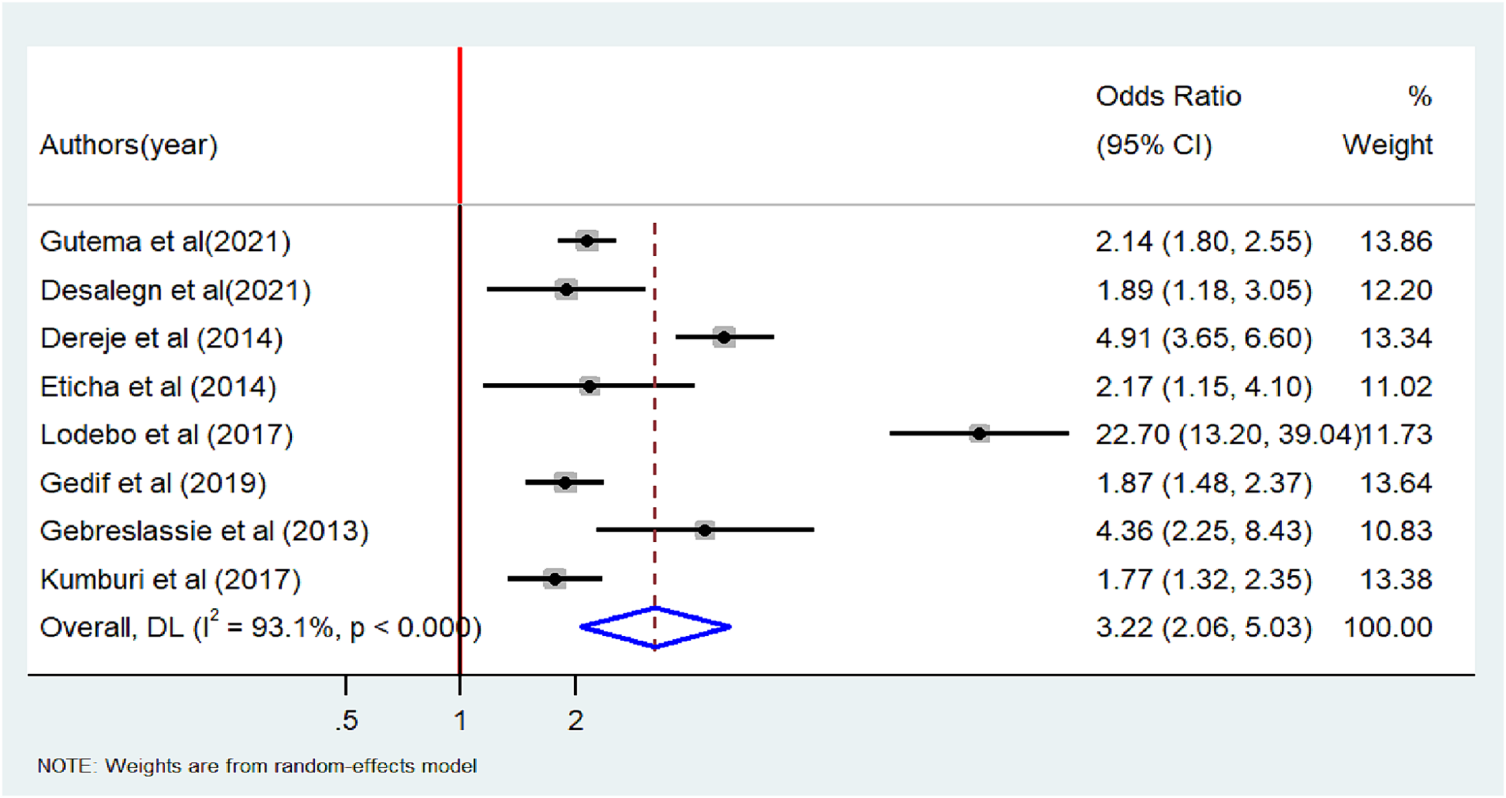
Forest plot of odds ratio for the association between male and tobacco smoking among adults in Ethiopia, 2023.

**Figure 8:**
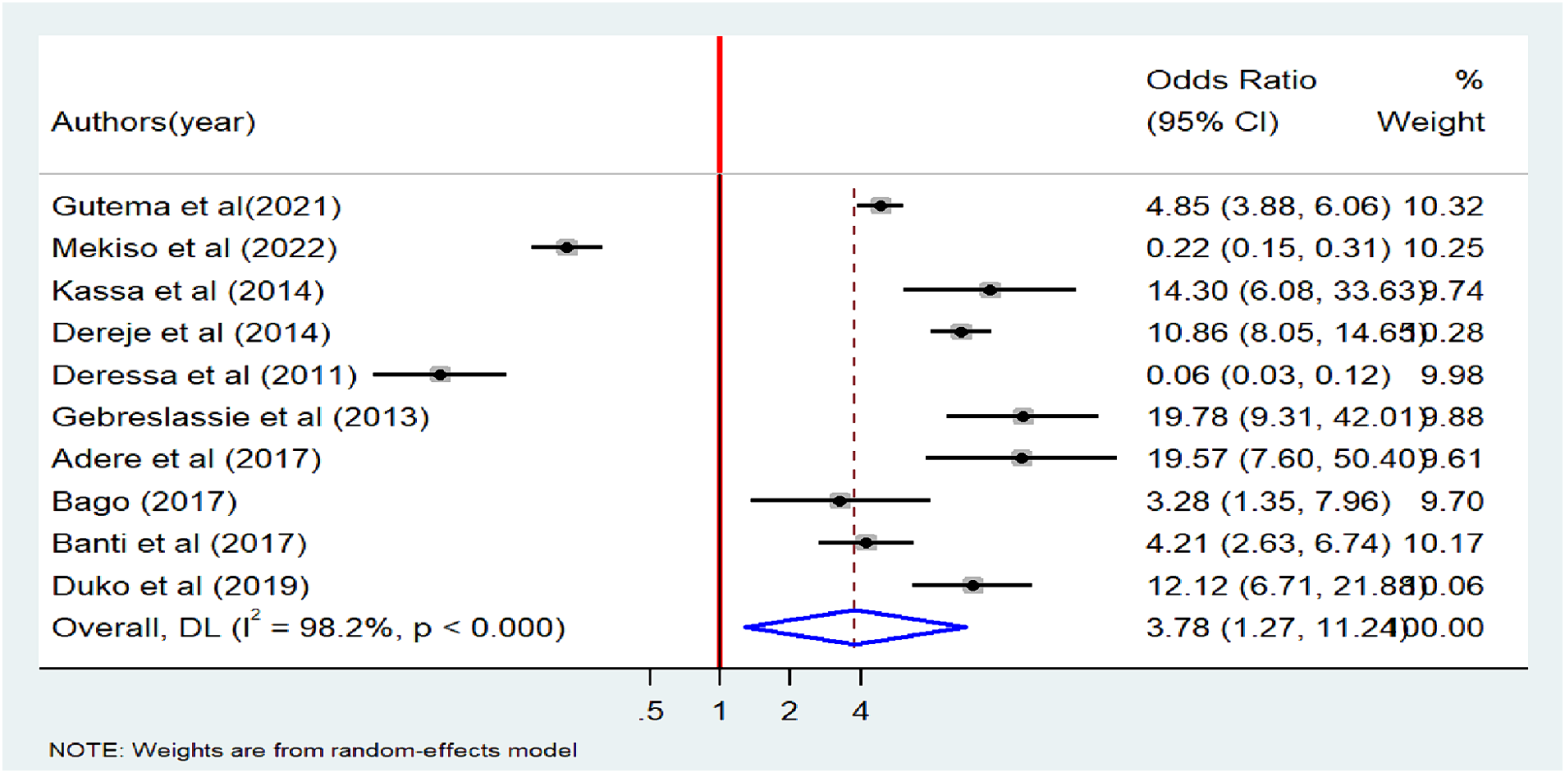
Forest plot of odds ratio for the association between alcohol use and tobacco smoking among adults in Ethiopia, 2023.

A total of 17 studies were used to identify factors associated with tobacco smoking (18–24, 26, 27, 29, 30, 34–36, 38, 39, 44). The results from the random-effects model showed that the odds of tobacco smoking were three times higher among males compared with females (OR = 3.22, 95% CI: 2.06–5.03). Significant heterogeneity was observed among the included articles (I^2^ = 93.1%, *p* < 0.001) (Figure 7). The association between alcohol consumption factors and tobacco smoking was examined based on the results of ten studies (18, 20–23, 26, 29, 31, 34, 39). Eight of the included studies had a positive association (18, 20–22, 26, 29, 31, 34) while a negative association was noted in the other two studies (23, 39). The odds of tobacco smoking were 3.78 times higher among alcohol users than non-alcohol users (OR = 3.78, 95%CI: 1.27–11.24) (Figure 8).

A total of 12 studies were included to identify the association between chewing *khat* and tobacco smoking (18, 20, 21, 23, 26, 27, 29, 31, 34, 36, 38, 44). Ten of the included studies had a positive association (18, 21, 26, 27, 29, 31, 34, 36, 38, 44) while a negative association was noted in the other two studies (20, 23). The result of the random-effects meta-analysis showed that non-significant association between tobacco smoking and chewing *khat* (OR = 3.96, 95%CI: 0.96–16.29) (Table 4).

**Table 4:** The pooled effect size of factors associated with lifetime tobacco smoking among adults in Ethiopia, 2023.

Variables	Number of study participants	The number of studies included	Odds ratio (95% CI)	Heterogeneity	
				*I*^2^ (%)	*p*-value
Male	9,383	8	2.31 (2.05–5.03)	93.1	<0.001
Education	6,165	4	1.25 (0.48–3.22)	96.9	<0.001
Alcohol use	9,651	10	3.07(1.27–11.24)	98.2	<0.001
Chewing *khat*	9,804	12	3.96 (0.96–16.29)	98.7	<0.001
Parental smoking	2,728	4	2.16 (0.28–16.83)	98.9	<0.001

**Figure 9:**
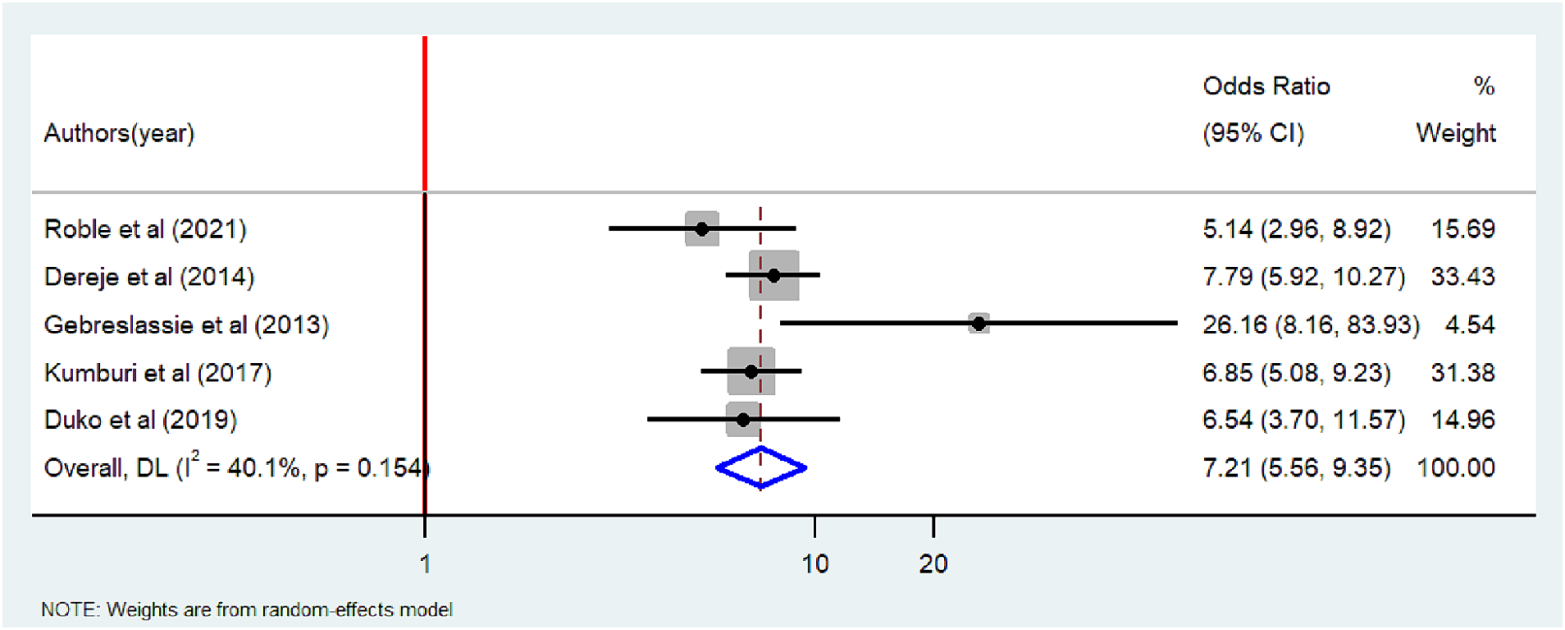
Forest plot of odds ratio describing having tobacco smoking friends and tobacco smoking among adults in Ethiopia, 2023.

Five studies (22, 26, 29, 36, 44) were included to assess if having tobacco-smoking friends is associated with tobacco smoking. All of the included studies had a positive association. The meta-analysis also showed that the odds of tobacco smoking were seven times higher among adults who have tobacco-smoking friends compared to those who did not have smoking friends [OR = 7.21 (95%CI: 5.56–9.35)], with extreme heterogeneity (I^2^ = 40.1%, *p* = 0.154) (Figure 9).

A total of 4 studies (22, 35, 39, 44) were included to assess if having a tobacco-smoking parent is associated with tobacco smoking. Three of the included studies had a positive association (22, 35, 44) while a negative association in one study (39). Meta-analysis also showed that there was a non-significant association between parental smoking habits and adult tobacco smoking [OR = 2.16 (95%CI: 0.28–16.83)] (Table 4).

## Discussion

Tobacco smoking continues to be a global public health problem and its burden increases over time, particularly in low- and middle-income countries. This systematic review and meta-analysis was conducted to estimate the pooled prevalence of tobacco smoking among adults in Ethiopia. The lifetime and current pooled prevalence of tobacco smoking among adults were 16.0 and 13.7%, respectively, with high heterogeneity. The figures in our report are greater than the findings from a meta-analysis conducted in Mainland China (10.8%) (59) and East Africa (9.0%) (60), which could be due to the lack of full enforcement and implementation of tobacco smoking in Ethiopia. It is documented that the lack of effective implementations of cigarette sale prevention policies contributes to the high proportion of tobacco smokers (61).

On the other hand, the prevalence in our study is lower than that reported in Saudi Arabia (28.1%) (62), Kenya (42.8%) (63), Poland (30.8%) (64), China (31.8%) (65), Madagascar (28.5%) (66), and Bangladesh (23.2%) (67). This difference could be due to variations in the study population and setting. For instance, most of the aforementioned studies were conducted among students, who were likely to be exposed to smoking as a result of their age and peer pressure. Evidence from our subgroup analysis also showed that the pooled prevalence of tobacco smoking was significantly higher among students than studies conducted among the general population, which is in agreement with previous reports from systematic review and meta-analyses (68).

Gender was a significant predictor of tobacco smoking. The odds of tobacco smoking were two times higher among males than females. This finding was in line with a report from systematic reviews done in Iran (69), Sub-Saharan Africa (70), Saudi Arabia (62), and a meta-analysis conducted among students in Ethiopia (68). This might be associated with better social acceptance of substance use, such as *khat,* tobacco, and alcohol, among males than females. On the other hand, family relationships including care and family-related activities may protect females from being involved in tobacco use (71).

The odds of tobacco smoking were three times higher among alcohol users than non-users. Our finding was in agreement with a previous report from the Nigerian community (72). This might be due to the fact that alcohol use often takes place in social settings where smoking is prevalent, especially in public places. Another possible reason for this finding might be due to peer influence and social norms that can enhance tobacco smoking behavior. Other studies also indicated that adults who drink alcohol are highly likely to smoke tobacco (73, 74). This implies that addiction invites further addictions.

The meta-analysis showed that the odds of tobacco smoking were seven times higher among adults who have smoking friends compared to those who did not have smoking friends. This finding was consistent with studies conducted in Nepal (75), Madagascar (76), Cameroon (77), and Jordan (78). This implies the need for continuous social influence programs that can help create social norms that discourage young people from starting to smoke.

### Strengths and limitations of the study

This review included most regions of the country and a diverse target population (students and other community members including healthcare workers and patients). The study followed preferred reporting items for systematic review and meta-analysis guideline. However, studies included in this systematic review and meta-analysis were cross-sectional, which limits the causality of predictors on tobacco smoking. Besides, this study included articles published in the English language only, which could induce publication bias.

## Conclusion

The pooled prevalence of tobacco smoking was high in Ethiopia. We found that one in six adults was engaged in tobacco smoking, which calls for urgent intervention. Being male and alcohol users, and having tobacco-smoking parents and friends were found to be factors positively associated with tobacco smoking. Therefore, urgent comprehensive public health intervention should be taken to reduce the burden of the problem. Moreover, the federal Ministry of Health, community leaders, and other concerned bodies should take strong action to protect the health of adults. In addition, alcohol control law enforcement should be strengthened.

## Data availability statement

The original contributions presented in the study are included in the article/supplementary material, further inquiries can be directed to the corresponding author.

## Author contributions

CD: Conceptualization, Data curation, Formal analysis, Funding acquisition, Investigation, Methodology, Project administration, Resources, Software, Supervision, Validation, Visualization, Writing – original draft, Writing – review & editing. AA: Conceptualization, Formal analysis, Investigation, Methodology, Validation, Visualization, Writing – review & editing. SD: Formal analysis, Investigation, Methodology, Software, Validation, Visualization, Writing – review & editing. MD: Data curation, Funding acquisition, Investigation, Resources, Supervision, Validation, Writing – review & editing. BD: Conceptualization, Investigation, Methodology, Resources, Supervision, Validation, Writing – review & editing. MG: Data curation, Investigation, Resources, Supervision, Validation, Visualization, Writing – review & editing.
